# Advancing aquaculture probiotic discovery via an innovative protocol for isolation of indigenous, heat and salt tolerant, quorum quenching probiotic candidates

**DOI:** 10.3389/fmicb.2025.1558238

**Published:** 2025-04-02

**Authors:** Philip Rwezawula, Wilson Waiswa Mwanja, Nick Vereecke, Peter Bossier, Daisy Vanrompay

**Affiliations:** ^1^Laboratory of Aquaculture & Artemia Reference Center, Department of Animal Science and Aquatic Ecology, Faculty of Bioscience Engineering, Ghent University, Ghent, Belgium; ^2^Busitema University Maritime Institute, Tororo, Uganda; ^3^Laboratory of Immunology and Animal Biotechnology, Department of Animal Production and Aquatic Ecology, Faculty of Bioscience Engineering, Ghent University, Ghent, Belgium; ^4^PathoSense BV, Lier, Belgium; ^5^Laboratory of Virology, Faculty of Veterinary Medicine, Ghent University, Merelbeke, Belgium

**Keywords:** aquaculture, quorum sensing, quorum quenching, N-hexanoyl homoserine lactones (HHLs), probiotics, stress tolerance, biosafety

## Abstract

**Introduction:**

The intensification of aquaculture to meet the growing demand for aquatic animal protein by a global population approaching 10 billion by 2050 has raised concerns about the increased risk of disease outbreaks in farmed species. These diseases account for over 50% of economic losses in commercial aquaculture, largely due to the reliance on ineffective and harmful therapeutic options like antibiotics, which contribute to multidrug resistance and pose serious global health concerns. However, non-antibiotic alternatives such as probiotics have emerged promising choices to enhance growth performance, immunity, and disease resistance in aquaculture species.

**Methods:**

In this study, we present a novel, non-invasive protocol for isolating indigenous bacteria from sediment. This method utilizes minimal media supplemented with N-hexanoyl homoserine lactone (HHL) to select strains with key probiotic attributes.

**Results and discussion:**

Our study isolated 24 bacterial isolates, 11 demonstrating quorum quenching (QQ) activity by degrading HHL, indicating potential for antivirulence therapy. Among these, eight were non-hemolytic, suggesting safety in the presence of host wounds. Six non-hemolytic isolates exhibited proteolytic activity, which is essential for aiding protein digestion. Whole genome sequencing revealed their identity as *Priestia megaterium* PMUG01 and PMUG02, *Lysinibacillus fusiformis* LFUG, *Micrococcus yunnanensis MYUG*, and two novel species tentatively named *Kocuria crassamentum species nova* strain KSNUG, and *Heyndrickxia crassamentum species nova* strain HSNUG. Despite some virulence-associated genes, none of the strains demonstrated pathogenicity in *Artemia* nauplii. Apart from an *lsaB* gene in *P. megaterium*, which confers resistance to lincosamides, no antibiotic resistance genes were detected. Our findings highlight these strains’ biosafety and probiotic potential for aquaculture, offering promising candidates for sustainable disease management and improved feed utilization in farmed species. These results pave the way for developing indigenous and effective, non-antibiotic-based probiotic solutions to mitigate disease risks in aquaculture.

## 1 Introduction

Pathogenic bacteria use quorum sensing (QS) to regulate virulence gene expression and enhance antimicrobial resistance (AMR) via cell-cell communication with autoinducers (AIs) such as N-acyl homoserine lactones (AHLs) (Defoirdt, 2018). The disruption of this QS mechanism, also known as quorum quenching (QQ), can inhibit virulence gene activation and alter bacterial infectious cycles, thereby controlling diseases caused by such pathogens (LaSarre and Federle, 2013). Unlike bactericidal antibiotics, QQ mechanisms do not significantly affect bacterial growth, minimizing selection pressure for AMR phenotypes, thus making it a promising antivirulence approach (Ghanei-Motlagh et al., 2021). As highlighted by Kuebutornye et al. (2019), certain groups of bacteria such as *Bacillus* spp., that have shown a high probiotic potential in aquaculture, exhibit these QQ properties. Probiotics are live microorganisms that offer various benefits such as enhanced immunity and resistance to pathogens (El-Saadony et al., 2021). With increasing concerns on the use of antimicrobial agents in aquaculture, probiotic bacteria emerge as a safer alternative (Lu et al., 2022; Serwecińska, 2020). In finfish aquaculture, most used probiotic bacteria belong to genera of *Lactobacillus*, *Lactococcus*, *Pediococcus*, and *Bacillus* (Kuebutornye et al., 2019; Yousuf et al., 2023). Formerly, probiotics used in aquaculture were allochthonous in nature, isolated from terrestrial sources, disregarding their inadequate efficacy for aquaculture due to physiological incompatibility between terrestrial and aquatic conditions within the hosts and their environments (Yousuf et al., 2023). However, consensually, probiotic strains isolated from aquatic organisms, or their environment colonize and establish themselves relatively faster, are more stable, resilient, persistent, and robust. Furthermore, host immune systems barely act on them as they are already part of the gut microflora (Nayak, 2010; Yousuf et al., 2023). Thus, indigenous QQ bacteria, isolated from aquatic hosts and their environments should already be adapted to local conditions (*e.g.*, tolerance to salt and high temperatures). This would offer inherent resistance to environmental stressors and may exhibit improved efficacy against local emerging aquaculture pathogens (Amenyogbe et al., 2022; Husain et al., 2022; Kaushik et al., 2009), including *Aeromonas* spp., *Pseudomonas* spp., *Edwardsiella* spp., *Flavobacterium* spp., *Mycobacterium* spp., *Streptococcus* spp., amongst others (Akoll and Mwanja, 2012; Walakira et al., 2014).

We explored indigenous reservoirs of bacteria from aquaculture production systems in Uganda, focusing on their antivirulence potential, environmental adaptability and compatibility for probiotic application in aquaculture. Our goal was to enhance preparedness for combatting aquatic pathogens and promote sustainable aquaculture productivity. This work focused on the development of an innovative protocol to aid in the discovery of putative new indigenous candidate probiotics for use in aquaculture. Our approach included procedures on selective bacterial isolation from aquaculture environments (i.e., sediments), phenotypic screening, molecular identification, and *in vivo* biosafety assessment. The primary objective of our study was to isolate indigenous bacterial strains exhibiting enhanced QQ capabilities and enhanced survival under elevated temperature (i.e., 85°C) and high salinity (i.e., 35 g.L^–1^ NaCl) conditions. This targeted approach allowed to obtain candidate probiotic strains that could be further exploited for their QQ-based antivirulence potential. Our approach also included heat and salt selective pressure to exclude virulent *Bacillus cereus* strains, known for their involvement in food poisoning incidents and thus of importance for public health concerns (Wang et al., 2020). Furthermore, to reassure the probiotic nature of these indigenous bacteria, a phenotypic screening was conducted with a focus on key traits relevant for their probiotic application. This included QQ rates, hemolysis, and proteolytic activity to select the most promising candidates for probiotic use in Ugandan aquaculture. Our protocol also included in-depth molecular analyses using whole genome sequencing (WGS) with Oxford Nanopore Technologies (ONT) for bacterial identification and genomic probiotic prediction. Finally, we assessed the biosafety of the strains in axenic brine shrimp (*Artemia franciscana*), following a previously established toxicity testing model (Roy et al., 2019).

## 2 Materials and methods

### 2.1 Sample collection and isolation of candidate probiotic bacteria

**FIGURE 1:**
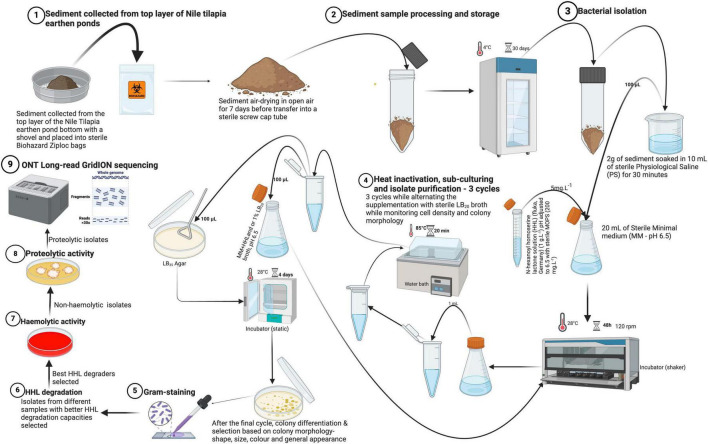
Overview of the isolation protocol for the isolates from sample collection, isolation, purification, selection of isolates to submission for molecular characterization (WGS). Physiological saline (PS) was composed of 8.0 g.L^– 1^ NaCl, 0.2 g.L^– 1^ KCl, 1.44 g.L^– 1^ Na_2_HPO_4_, and 0.24 g.L^– 1^ KH_2_PO_4_. Minimal medium (MM) contained 0.080 g.L^– 1^ NaHCO_3_, 0.250 g.L^– 1^KCl, 0.040 g.L^– 1^KBr, 1.840 g.L^– 1^ MgCl_2_6.H_2_O, 0.410 g.L^– 1^ CaCl_2_.2H_2_O, 0.008 g.L^– 1^ SrCl_2_.6H_2_O, and 0.008 g.L^– 1^ H_3_BO_3_ (Created with BioRender.com).

Wet sediment samples were collected with a plastic shovel from the surface layer of Nile tilapia (*Oreochromis niloticus*) earthen ponds at Uganda’s National Aquaculture Research and Development Centre (ARDC), part of the National Agricultural Research Organization (NARO). Sediment samples were airdried in open air on sterile plastic petri dishes until they were completely dry (∼7 days). They were then packed in sterile air-tight Ziploc bags (Biohazard, China) and stored at 4°C for 30 days until they were used to isolate native bacteria. Samples were subjected to our bacterial isolation protocol to select for quorum quenching (QQ) and stress tolerance (i.e., heat at 85°C and NaCl at 35 g.L^–1^). This part of the protocol is thought to benefit the elimination of virulent *Bacillus cereus*. A schematic overview of the bacterial isolation protocol can be found in Figure 1. Firstly, minimal medium [MM; 0.080 g.L^–1^ NaHCO_3_, 0.250 g.L^–1^KCl, 0.040 g.L^–1^KBr, 1.840 g.L^–1^ MgCl_2_6.H_2_O, 0.410 g.L^–1^ CaCl_2_.2H_2_O, 0.008 g.L^–1^ SrCl_2_.6H_2_O, and 0.008 g.L^–1^ H_3_BO_3_ in phosphate buffered saline (pH 6.5)] was prepared and autoclaved. Also, a 50 mg.mL^–1^ N-hexanoyl homoserine lactone (HHL; fluka, Germany) stock solution was prepared by dissolving 10 mg of HHL in 200 μL of ethanol (95%), followed by filter-sterilization using 0.22 μm Polyvinylidene fluoride (PVDF) syringe filters (30 mm diameter; Whatman). The final stock solution (1 g.L^–1^) was obtained by dilution with sterile distilled water and stored at 4°C until use.

For the actual bacterial isolation, 2 g of each sediment sample was soaked for 30 min in 10 mL of a sterile physiological solution (PS; 8.0 g.L^–1^ NaCl, 0.2 g.L^–1^ KCl, 1.44 g.L^–1^ Na_2_HPO_4_, and 0.24 g.L^–1^ KH_2_PO_4_). From this 10 mL suspension, 100 μL was dispensed into 20 mL of MM supplemented with buffered [pH adjusted to 6.5 with sterile 3-(N-morpholino) propanesulfonic acid) (MOPS) (200 mg.L^–1^) before storage] HHL to a final concentration of 5 mg.L^–1^ as a sole source of nitrogen and carbon. Incubation of the samples was done in sterile glass tubes at 28°C for 48 h on a shaker (BIOSAN ES-20) at 120 rpm. After initial incubation, 1 mL of each sample was aliquoted into sterile 1.5 mL microcentrifuge Eppendorf tubes and heat-treated for 20 min at 85°C in a water bath to further minimize the likelihood of (co-) isolating toxin producing *Bacillus cereus*. After heat-treatment, 100 μL of the cultures were spread on sterile Luria Bertani 35 (LB_35_; LB with 35 g.L^–1^ NaCl) agar plates and incubated for 4 days at 28°C with continuous monitoring of colony formation and concentration (CFU.mL^–1^). Thereafter, 100 μL from each of the remaining heat-treatments was inoculated into new sterile MM, supplemented with buffered HHL and 1% sterile Luria Bertani 20 (LB_20_; LB with 20 g.L^–1^ NaCl) broth for another incubation cycle. Plating was done on LB_35_ agar plates to screen for strains with a high salt tolerance capability. Alternating the addition of LB_20_ broth (1%) to MM aimed to increase nutrient availability for the selected strains and enhance their concentration through the subsequent cycles of isolation and purification. Heat-treatment and growth on LB_35_ agar plates, each time followed by inoculation of colonies in MM supplemented with buffered HHL (5 mg.L^–1^) and/or 1% LB_20_ broth was repeated for three cycles. Thus, a total of three sub-cultures were made to purify and select distinct colonies on LB_35_ agar for further characterization and screening. After the final cycle in MM, single colonies on LB_35_ agar were selected based on morphological differences (i.e., shape, color, size, and general appearance). All isolates were Gram-stained according to Paray et al. (2023) and all candidate probiotic isolates were cryopreserved in 50% sterile glycerol stocks and stored at −80°C.

### 2.2 *In vitro* screening of candidate probiotics

#### 2.2.1 Assessing the ability of quorum quenching by measuring HHL degradation

Due to the importance of QQ in controlling bacterial virulence in aquaculture, the ability for QQ was assessed for our candidate probiotic isolates. We applied a protocol involving a plate diffusion method to quantitatively detect exogenous HHL degradation by QQ isolates to aid in the selection of QQ strains as described by Defoirdt et al. (2011). This method involves detecting exogenous AHLs, which are QS autoinducers, using *Chromobacterium violaceum* strain CV026. This strain does not produce AHLs but responds to their presence by producing violacein, a visible purple pigment (Defoirdt et al., 2011; Tinh et al., 2007).

In short, candidate probiotic isolates were inoculated on Luria Bertani 10 (LB_10_; LB with 10 g.L^–1^ NaCl) agar and after overnight incubation at 28°C, a single colony was inoculated into 5 mL of buffered LB_10_ broth (pH 6.5 with MOPS, 200 mg.L^–1^), supplemented with 5 mg.L^–1^ HHL. Incubation was done at 28°C and 120 rpm for 24 h. This procedure was repeated for three independent colonies to obtain triplicate biological read-outs. Simultaneously, the *C. violaceum* CV026 reporter strain was grown overnight (28°C, shaking at 120 rpm) in LB_10_ broth buffered to pH 6.5 with sterile MOPS and supplemented with kanamycin (20 mg.L^–1^). The addition of kanamycin warrants selective growth of CV026 by suppressing potential contaminants and to promote retention and maintenance of the plasmid that enables the strain to detect exogenous AHLs (Devescovi et al., 2017; Kroll et al., 2010). Besides our isolates, negative and positive control strains, *Pseudomonas flourescens* pME6000 and pME6863, being AHL degrading and AHL non-degrading strains, respectively, were included to guarantee valid assay read-outs (Defoirdt et al., 2011; Gopu and Shetty, 2016). To detect HHL degradation by our candidate probiotic isolates, the OD_550_ of *C. violaceum* CV026 was adjusted to 0.1 before making lawns on LB_10_ agar plates with 100 μL of the culture. For the standard curve, 10 μL from each HHL dilution series was spotted in the center on the *C. violaceum* CV026 lawns in triplicate and incubated at 28°C for 24 h (Supplementary Figure S1). For the test isolates and controls, 1 mL was aliquoted after 24 h of incubation, filter-sterilized (0.22 μm PVDF syringe filter, diameter 30 mm, Whatman) to obtain bacteria-free supernatants, and spotted (10 μL) in the center onto *C. violaceum* CV026 lawns. LB_10_ plates with *C. violaceum* CV026 lawns containing spots were incubated for 48 h at 28°C and the diameters of the purple halos of the violacein pigment on CV026 lawns were measured with a ruler (300 mm) and correlated with the standard curve (Supplementary Figure S1). A linear regression model was then fitted to estimate HHL concentrations during the HHL degradation experiments for the test isolates and controls. Isolates capable of significantly reducing HHL concentration below the negative control level were classified as HHL degrading (i.e., QQ isolates) and were retained for further analysis.

#### 2.2.2 Visualization of *in vitro* QQ

The selected isolates were cross streaked with an AHL producer (i.e., *Aeromonas hydrophila* strain LVS3), and an AHL reporter (*C. violaceum* strain CV026) parallel to each other at a separation not exceeding 15 mm. All the test strains and the negative control (sterile LB_10_ broth) were streaked at the center between the parallel cross streaks of *A. hydrophila* LVS3 and *C. violaceum* CV026. The QQ capability of our test isolates was confirmed when purple pigment formation occurred only where the *C. violaceum* CV026 streak was unobstructed by the QQ positive isolates as compared to the negative control. The latter is characterized to not inhibit purple pigmentation along the *C. violaceum* CV026 streak.

#### 2.2.3 Hemolytic activity of candidate probiotics

Assessing hemolytic activity is crucial for probiotic biosafety as hemolytic bacteria can profit from small lesions and wounds on the skin of hosts and cause infections (Jinendiran et al., 2019). Therefore, selected QQ-confirmed isolates were evaluated further for their hemolytic activity as described by Kuebutornye et al. (2020). Briefly, all isolates were tested for hemolysis on LB_10_ agar plates supplemented with 5% (v/v) defibrinated sheep blood. The OD_550_ of the overnight grown cultures of the isolates was adjusted to 0.1 before spotting 5 μL of each isolate onto the center of the plates in triplicate, followed by incubation at 28°C for 48 h. Isolates which induced complete hemolysis (β-hemolytic) were identified by clearance around and below the colony spots while isolates which induced partial hemolysis (α-hemolytic) exhibited greenish-brownish zones around and below the colony spots. Non-hemolytic isolates (^ɣ^-hemolytic) were identified by the absence of clearance around the colony spots. Isolates that displayed β-hemolysis were eliminated, while those that exhibited ^ɣ^ or α hemolysis were considered safe and were retained for further screening and molecular characterization (Kuebutornye et al., 2020).

#### 2.2.4 Proteolytic activity of candidate probiotics

Proteolytic activity is essential for protein utilization, the most expensive nutrient in fed aquaculture production (Liu et al., 2009). Hence, all QQ-confirmed and ^ɣ^ or α hemolytic isolates were subjected to an assay to assess their proteolytic activity by streaking them on skim milk agar plates (10% w/v) followed by incubation at 28°C for 72 h in triplicate (Hossain et al., 2021). Isolates positive for proteolysis as indicated by the presence of clearance zones on the skim milk agar plates were selected for further molecular characterization using Oxford Nanopore Technologies’ (ONT) long-read whole genome sequencing (WGS) and subsequent *in vivo* biosafety testing.

#### 2.2.5 Long-read whole genome sequencing, species identification, and *in silico* evaluation of probiotic activity and potential risks

All Gram-positive, *in vitro* confirmed QQ, ^ɣ^ or α hemolytic, and proteolytic isolates were revived from a freezer (-80°C) and cultured overnight at 28°C on LB_10_ agar plates. These plates were subsequently transported to the PathoSense laboratory (Merelbeke, Belgium), where WGS was conducted using the ONT long-read GridION sequencing platform, according to Vandeputte et al. (2024) and Vereecke et al. (2023). Final genome assemblies were submitted to NCBI under BioProject PRJNA1094437 with accession numbers as represented in Supplementary Table S2. Resulting genome assemblies were identified and classified at the species level using rMLST (pubMLST) (Jolley et al., 2012) and the Type Strain Genome Server (TYGS) (Meier-Kolthoff and Göker, 2019). New bacterial species were assessed using TYGS dDDH (d4) along with average nucleotide identities (ANI) as obtained from the ANI calculator on EZBioCloud (Yoon et al., 2017). A new species was determined based on dDDH (d4) and ANI values below 70 and 95%, respectively (Goris et al., 2007; Meier-Kolthoff and Göker, 2019). Next, we used our genomes to perform an *in silico* prediction and assessment of their probiotic nature and safety using two new tools. A first approach, iProbiotics (v.2023.6.5) (Sun et al., 2022) uses machine learning for the rapid identification of probiotic properties. Here, the “model1: Probiotic Predictor” was used for probiotic prediction. Our analysis was supplemented with well-known and relevant bacterial probiotics and pathogens as controls based on Kuebutornye et al. (2019) (Supplementary Table S3). Secondly, our genomes were submitted to the ProBioMinServer (Liu et al., 2023), an integrated platform to assess the safety and functional properties of putative prokaryotic strains. This approach delivered a Probiotic Potential Risk Score (PPRS) as the overall sum of the presence of Antimicrobial Resistance Genes (ARGs), virulence factors, and Mobile Genetic Elements (MGEs), which is suggested to be interpreted as low-risk (≤4), medium-risk (4–6), and high-risk (≥6). An overview of the results, used software packages, their versions, and databases is given in Supplementary Table S4. Finally, to reassure ourselves from the rigor of these tools, independent screenings against the CARD (Alcock et al., 2023) and complete VFDB (Liu et al., 2019) databases were conducted using Abricate (v.0.9.9).^1^ This allowed us to analyze the data in “conserved” and “loose” mode, representing 80%/80% and 60%/60% for nucleotide identity and query coverage, respectively. Complete data output is represented in Supplementary Table S5.

### 2.3 *In vivo* biosafety/non-toxicity verification

To validate the biosafety and thus non-toxicity of the molecularly characterized strains, axenic brine shrimp (*Artemia franciscana*) nauplii (instar II stage) were bath-treated with the bacterial strains at a cell density of 10^7^ CFU.mL^–1^ according to Roy et al. (2019) with minor modifications. *Artemia* cysts were first decapsulated and then suspended in filtered and autoclaved artificial sea water (FAASW—35 g.L^–1^ instant Ocean synthetic sea salt, Aquarium Systems, Sarrebourg, France). The cysts were incubated at 28°C for 24 h on a rotor (6 rpm) until hatching. The nauplii were incubated (2 nauplii per mL) in sterile glass tubes containing FAASW (10 mL) on a rotor (28°C, 6 rpm, 48 h). Bacterial strains were prepared by inoculating overnight cultures into sterile LB_10_ broth and harvested them at the log phase. Cultures were centrifuged (MF 20-R, France) (7,000 × g, 4°C, 15 min) to obtain pellets, washed three times, and resuspended in sterile FAASW (10 ppt) to make stocks. Optical densities at 550 nm were measured to estimate the cell density for bath treatments. Nauplii were fed once with autoclaved *A. hydrophila* LVS3 at a concentration of 10^8^CFU.mL^–1^. Cell densities were estimated using a McFarland standard (BioMerieux, Marcy L’Etoile, France) at 550 nm, assuming that OD550 of 1 corresponds to 1.2 × 10^9^ Cells.mL^–1^. After 48 h, survival rates computed and compared between test groups and mock infections. A significant reduction in survival rates in the test groups compared to the negative controls indicated potential toxicity of the bacterial strains, while similar survival rates confirmed their biosafety.

### 2.4 Data analysis and statistics

The QQ potential between the test isolates and the controls was compared by an Independent Two sample *t*-test in which significance was determined if *p* < 0.05. For the HHL degradation assay, a correlation between the diameters of the purple halos and the HHL concentrations in the HHL dilution series was used to compute a standard curve. A linear regression equation from the curve was used to calculate the estimated HHL/AHL concentration for the different isolates. *In vivo* survival rate data for the biosafety trial were first transformed (i.e., arcsine) to fulfill the requirements of normality and homoscedasticity prior to running a One-way ANOVA in which significance was determined if *p* < 0.05. A Tukey’s HSD *post-hoc* test was done for multiple comparison at 95% confidence interval (CI). Statistical analysis was conducted in R studio (v. 2023.03.0 + 386). Graphs were produced by Microsoft excel, GraphPad Prism (version 9, GraphPad Software, San Diego, CA, United States), and Sigma plot (v.15).

## 3 Results

### 3.1 Isolation and QQ-based screening of indigenous probiotic candidates

**FIGURE 2:**
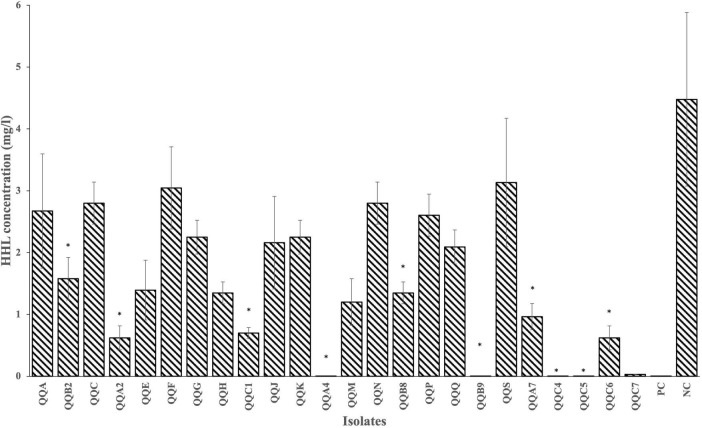
Results of the plate diffusion assay. HHL degradation by all 24 Gram-positive isolates was measured after 24 h (28°C) of incubation in LB_10_ broth supplemented with 5 mg.L^– 1^ buffered HHL. PC and NC denote the positive (*P. flourescens* pME6863) and negative (*P. flourescens* pME6000) controls, respectively.

**FIGURE 3:**
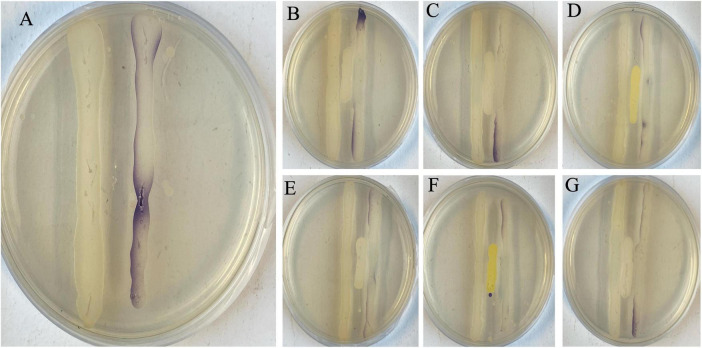
Visualization of *in vitro* quorum quenching. (**A**) Negative control (LB_10_ broth), streaked in between an AHL producer (LVS3) and an AHL reporter (CV026 – purple streak). The whole streak of CV026 was filled with a purple pigment after detecting the AHLs from LVS3 without degradation. All the selected strains (**B** – PMUG01, **C** – PMUG02, **D** – MYUG, **E** – LFUG, **F** – KSNUG and **G** – HSNUG) were streaked in between the AHL producer (LVS3) and AHL reporter (CV026) and absence of the purple pigment on the CV026 streaks in zones where the strains were streaked indicated AHL degradation by the QQ strains.

From nine sediment samples, 24 Gram-positive isolates were selected based on their diverse morphological characteristics (i.e., size, color, shape, and overall appearance) as summarized in Supplementary Table S1. All the 24 isolates were tested for their HHL degrading capacity with a plate diffusion assay. The concentration of HHL (in mg.L^–1^) was determined using the equation derived from the HHL standard curve obtained from the linear regression on the standard curve (y = 1.2133x + 3.0952, *R*^2^ = 0.992). The negative control strain (*P. flourescens* pME6000) gave no HHL degradation (4.4775 ± 1.4027), while the positive control strain (*P. flourescens* pME6863) did (0.0005 ± 0.0000) (Supplementary Table S1). Eleven of the 24 isolates (QQB2, QQA2, QQC1, QQA4, QQB8, QQB9, QQA7, QQC4, QQC5, QQC6, QQC7) showed the ability to degrade HHL to concentrations significantly lower (*p* < 0.05) than the one observed for the negative control strain *P. flourescens* pME6000 (Figure 2; Supplementary Table S1). During QQ visualization, no AHL degradation was observed on the negative control plate (LB streak) (Figure 3A). In contrast, all the isolates effectively degraded AHLs produced by the AHL producer strain LVS3, represented by the selected strains (Figures 3B–G), reducing AHL concentrations to undetectable levels by the AHL reporter strain (CV026 streak). These strains were designated as QQ-positive and subjected to further analysis.

### 3.2 Hemolytic and proteolytic activity of candidate probiotics

Since all 11 test strains were confirmed to be QQ, they all underwent further evaluation for their hemolytic activity. This was done to align with biosafety considerations to determine their suitability as potential probiotic strains for application in aquaculture. While three isolates demonstrated hemolytic activity (β-hemolysis—QQB8, QQB9, and QQC4), three (isolates QQA2, QQB2, and QQA7) and five (isolates QQC1, QQC5, QQC6, QQC7, and QQA4) isolates were ^ɣ^- and α-hemolytic, respectively (Table 1). As such, the β-hemolytic isolates were excluded from further consideration, whereas the remaining eight isolates were subjected to further testing. These eight residual strains were subjected to an assay to assess their proteolytic activity to confirm and support their putative probiotic character as this is in line with enhanced protein utilization. A total of six (QQA2, QQB2, QQC1, QQA4, QQC5, and QQC6) isolates exhibited proteolytic activity and were subjected to subsequent molecular characterization by long-read WGS (Table 1).

**TABLE 1:** Overview of QQ confirmed putative probiotic isolates (*n* = 11) from aquaculture sediments, comprising *in vitro* QQ rate (in%), hemolytic activity and proteolytic activity of the isolates.

Isolate	*In vitro* QQ rate (% ± SD)	Hemolysis	Proteolysis	Probiotic suitability
QQB2	68.4 ± 7.7	ɣ	++	Yes
QQA2	87.6 ± 4.4	ɣ	++++	Yes
QQC1	86.0 ± 2.0	α	++	Yes
QQA4	100.0 ± 0.0	α	+++	Yes
QQB8	76.0 ± 8.4	β	NA	No
QQB9	100.0 ± 0.0	β	NA	No
QQA7	80.7 ± 4.7	ɣ	–	No
QQC4	100.0 ± 0.0	β	NA	No
QQC5	100.0 ± 0.0	α	+++	Yes
QQC6	87.6 ± 4.4	α	+++	Yes
QQC7	99.4 ± 0.1	α	–	No
PC	100.0 ± 0.0	NA	NA	NA
NC	10.5 ± 1.4	NA	NA	NA

The three β-hemolytic isolates were considered unsafe and were not tested for proteolytic activity (NA: Not Assessed). Isolates with significantly higher QQ rate than the negative control, non-hemolytic (α and ^ɣ^) and proteolytic in nature were considered suitable (Yes) for probiotic application in aquaculture whereas those that did not satisfy the above criteria were not (No). Relative proteolytic activity was ranked as ++++ (highest), followed by +++, and ++.

### 3.3 Identification and classification of selected isolates

Following ONT long-read sequencing and genome assembly, complete circular genome assemblies for the six remaining isolates were obtained that were evaluated to be suitable (i.e., QQ confirmed, non-hemolytic (^ɣ^ and α hemolysis), and proteolytic positive; Table 1) as probiotics in aquaculture. A complete overview of raw read QC, assembly QC, and genome identification can be found in Supplementary Table S2. Two new bacterial species, *Kocuria crassamentum species nova, strain KSNUG and Heyndrickxia crassamentum species nova, strain HSNUG* were identified (dDDH (d4 in%) ≤ 70% and ANI ≤ 95%).

**FIGURE 4:**
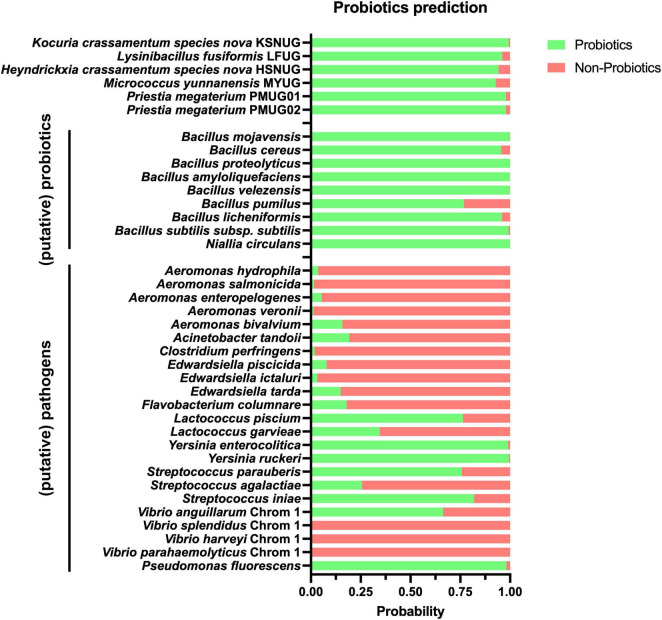
Results of the iProbiotics analyses using the machine learning model for probiotic prediction. The probability of our strains (n = 6) and a selection of relevant (putative) probiotics (n = 9) and (putative) pathogens (n = 23) are shown, with their probability of prediction to be a probiotic and non-probiotic in green and red, respectively. Selection of (putative) probiotics and pathogens is based on Kuebutornye et al. (2020). A detailed break-down is given in Supplementary Table S3.

Next, our genomes were used to assess their putative role as probiotics using the iProbiotics tool, which relies on machine learning to rapidly identify probiotic properties. As shown in Figure 4, all strains were classified as probiotic with a probability ranging between 92 and 99%, which is in correspondence with (putative) probiotics (96.3 ± 3.7% probiotic prediction probability; *n* = 9) and in contrast with (putative) pathogens (67.4 ± 37.3% non-probiotic prediction probability; *n* = 23) in aquaculture. Of note, the lowered non-probiotic prediction probabilities were a result of higher probiotic prediction probabilities for *Lactococcus piscium*, *Yersinia enterolitica*, *Yersinia ruckeri*, *Streptococcus iniae*, and *Pseudomonas fluorescens*. Removing these from the analysis resulted in a non-probiotic prediction probability of 83.6 ± 22.3% for the remaining (putative) pathogens (*n* = 18) (Supplementary Table S3). Second, a more extended approach was applied to assess the safety of our potential probiotics using the ProBioMinServer platform, which delivers a Probiotic Potential Risk Score (PPRS). Based on their scoring system, only two of the six putative probiotic strains (*Kocuria crassamentum species nova*, strain KSNUG and *M. yunnanensis* MYUG) were classified as low-risk (PPRS ≤ 4). The *L. fusiformis* LFUG, *Heyndrickxia crassamentum species nova* HSNUG, and *P. megaterium* PMUG01/PMUG02 strains were all classified as high(er) risk with PPRSs of 6.08, 9, and 11, respectively (Supplementary Table S4). Genome mining for the presence of known ARGs in “conserved” mode (i.e., 80% nucleotide identity and query coverage) revealed only the *lsaB* gene in the *P. megaterium* genomes (Supplementary Table S5). Performing the same analysis for known virulence factor genes, three putative virulence factors were identified. These included the sphaericolysin gene (Bsph_4094 from *Lysinibacillus sphaericus* C3-41 (VFG043991) in the *L. fusiformis* LFUG strain, and genes encoding for an isocitrate lyase [*icl* from *Mycobacterium avium* H37Rv (VFG009263)] and a chaperonin *GroEL* [Rv0440 from *Mycobacterium tuberculosis* H37Rv (VFG043550)] in both the *Kocuria crassamentum species nova* KSNUG and *M. yunnanensis* MYUG strains. Extending our search to “loose” mode (i.e., 60% nucleotide identity and query coverage), the same genes were identified as reported above, along with the identification of extra ARGs and virulence factors as shown in Supplementary Table S5.

### 3.4 *In vivo* biosafety/non-toxicity verification

**FIGURE 5:**
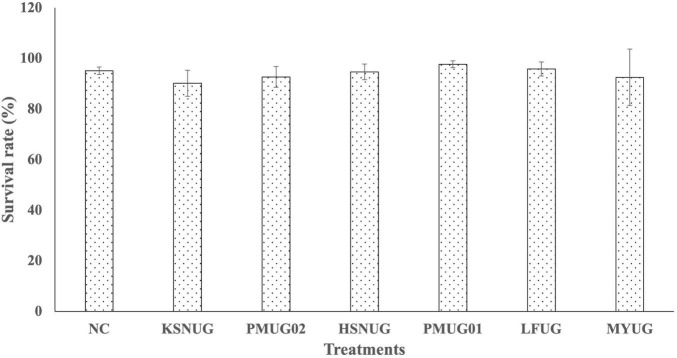
Survival rates of gnotobiotic *Artemia franciscana* nauplii after bath treatment with the selected candidate probiotic isolates in filtered and autoclaved artificial seawater (FAASW) and incubated for 48 h at 28°C on a rotor (6 rpm). The negative control (NC) was *Artemia* in FAASW, but without bacterial isolates. Survival rates in all groups were statistically the same.

The results of our genomic identification suggested that some strains might be of higher risk based on (i) ProBioMinServer PPRS scoring and (2) the identification of some putative virulence factors. Hence, we evaluated the biosafety of our six remaining isolates using an *in vivo* axenic *Artemia nauplii* (instar II) challenge and toxicity model. All isolates were shown to be non-toxic to *Artemia nauplii* (instar II stage) at 10^7^CFU.mL^–1^ after 48 h of incubation at 28°C (Figure 5). The survival rates for all treatments with the different strains were not significantly different from each other and from the negative control (*p* = 0.0624).

## 4 Discussion

Probiotics have caught substantial attention as non-antibiotic alternatives in aquaculture due to their ability to improve gut microbial balance, feed utilization, disease resistance, and overall health (Kuebutornye et al., 2020; Mathan Muthu et al., 2024). However, most probiotic isolation protocols prioritize *in vitro* antimicrobial activity, neglecting critical parameters such as host compatibility, colonization ability, and biosafety (Hasan and Banerjee, 2020). This limitation often leads to ineffective *in vivo* performance and potential disruptions to non-targeted microflora (Knipe et al., 2021). Consequently, molecular characterization, including virulence genes and antibiotic resistance gene (ARG) screening, is recommended for probiotic selection (Butt et al., 2024). Many commercial probiotics in aquaculture are allochthonous, exhibiting poor adaptability and colonization within aquatic hosts (Azad and Al-Marzouk, 2008; Yamashita et al., 2020). They may introduce foreign microbes into aquatic systems, where their interactions with resident microbiota are poorly understood (Azad and Al-Marzouk, 2008; Mukherjee et al., 2017; Villegas-Plazas et al., 2022; Yamashita et al., 2020). Therefore, isolation protocols should incorporate comprehensive molecular characterization and screening for robust, stress-tolerant, indigenous autochthonous strains.

In Uganda, fish health and biosecurity measures are underdeveloped despite the rise of intensive production systems such as cages, increasing the risk of disease outbreaks (Akoll and Mwanja, 2012; Mbowa et al., 2017; Walakira et al., 2014). Some probiotic strains have been isolated using classical *in vitro* techniques but lack thorough biosafety and molecular evaluation (Kato et al., 2016). Farmers have even added unknown microbes as probiotics to improve water quality (Naigaga, 2018). To address these gaps, our study developed a novel, non-invasive probiotic isolation pipeline focusing on stress-tolerant, autochthonous strains. We applied quorum quenching (QQ) and selective stressors (i.e., with heat at 85°C and salt at 35 g.L^–1^) to minimize co-isolation of virulent *B. cereus* while ensuring salt tolerance for stability in hosts and various aquatic environments (Berna et al., 2015). Noteworthy, using synthetic HHL in our protocol, and molecular characterization could hinder large-scale application.

Using our protocol on nine sediment samples, we obtained 24 Gram-positive bacterial isolates, 11 of which were QQ positive. Since quorum sensing (QS) regulates virulence in Gram-negative bacteria, QQ bacteria have potential as anti-virulence probiotics (Ghanei-Motlagh et al., 2021). Additionally, our approach reduced the likelihood of isolating *B. cereus* strains, which pose public health risks due to their involvement in food poisoning incidents (Wang et al., 2020). Given the spore-forming ability of *Bacillus cereus* strains, additional characterization of the isolates was necessary (Luu-Thi et al., 2014). In-depth screening (e.g., hemolytic activity and WGS) in our protocol, effectively excluded these strains from subsequent selection and use (Bhunia, 2018; Visiello et al., 2016). Specifically, all β-hemolytic isolates (03), comprising *B. cereus* and most fish and shellfish pathogens such as *Vibrio* spp., *Aeromonas* spp., *Flavobacterium* spp., *Edwardsiella* spp., *Streptococcus* spp., and *Mycobacterium* spp. were excluded (El-Saadony et al., 2021; Deng et al., 2021; Deng et al., 2023; Sarkar et al., 2021). The remaining eight isolates, classified as ^ɣ^ or α hemolytic, were considered safe for probiotic application (Kuebutornye et al., 2020).

To assess probiotic functionality, we evaluated extracellular enzyme production, particularly proteolytic activity, which enhances protein utilization in aquaculture (Cai et al., 2019; Khan et al., 2019). Six of the eight strains showed proteolytic activity and were further characterized using ONT long-read WGS. Whole genome sequencing and analysis confirmed pure isolates. These enabled precise species identification, highlighting the role of molecular identification and characterization in steering probiotic discovery toward strains with known specific probiotic attributes without biosafety concerns (Chau et al., 2021). Also, WGS revealed four *Bacillaceae* strains (i.e., HSNUG, PMUG01 and PMUG02, and LFUG), commonly used as aquaculture probiotics due to their beneficial traits such as the ability to: downregulate virulence gene expression in hosts; produce bacteriocins and antibiotics; compete for binding sites and nutrients with pathogen; synthesize essential substrates such as essential amino acids and vitamins; and possess immuno-modulatory and stimulatory properties in hosts (Kuebutornye et al., 2020). Their production of heat-resistant spores ensures prolonged storage viability and sustained effectiveness during practical application and administration (Kavitha et al., 2018; Nemutanzhela et al., 2014). Our new isolation protocol also discovered two novel bacterial species from *Kocuria* and *Heyndrickxia* genera, expanding potential probiotic candidates beyond *Bacillus* spp.

Accurate species classification required supplementation with external genomic databases (e.g., NCBI) due to incomplete public repositories (Goris et al., 2007; Meier-Kolthoff and Göker, 2019). For instance, the *M. yunnanensis* MYUG strain was initially misclassified as *M. luteus* due to database limitations.

The strains’ probiotic potential was even predicted further with advanced state-of-the-art computational tools such as iProbiotics and ProBioMinServer. A high probiotic potential (92–99%) was predicted. However, misclassifications for some of the included (putative) pathogens (e.g., *Lactococcus piscium*, *Yersinia enterolitica*, *Yersinia ruckeri*, *Streptococcus iniae*, and *Pseudomonas fluorescens*) suggest that these models were probably only trained on common (human) pathogens. Hence, refinement for aquaculture-specific strains is highly recommended.

Further biosafety analysis identified minimal ARGs and virulence factors, with only a few genes of uncertain functional significance detected at a relaxed (“loose”) threshold. Importantly, *in vivo* safety was confirmed in an axenic *Artemia* nauplii (instar II) challenge and toxicity model, where no significant mortalities occurred at 10^7^ CFU.mL^–1^ after 48 h of bath-treatment with the strains. Integrating *in vitro*, *in vivo*, and genomic assessments reinforced our selected strains’ safety and probiotic potential for aquaculture applications.

## 5 Conclusions

Our protocol prioritized the selection of six local, non-hemolytic and proteolytic stress-tolerant bacterial strains with enhanced quorum quenching capabilities out of 24 initially isolated strains, while minimizing the likelihood of co-isolating virulent strains such as *B. cereus*. These six strains were identified as *Priestia megaterium* PMUG01 and PMUG02, *Lysinibacillus fusiformis* LFUG, *Micrococcus yunnanensis* MYUG, and two novel strains, tentatively named *Kocuria crassamentum species nova* KSNUG and *Heyndrickxia crassamentum species nova* HSNUG. They all exhibited promising attributes for probiotic application in aquaculture from phenotypic, genomic, and *in vivo* biosafety perspectives. However, further studies are to be conducted to further understand their *in vivo* probiotic efficacy in different farmed organisms and at different life stages and augment their potency as non-antibiotic alternatives for aquaculture. Moreover, our protocol can be exploited further for its application on other samples of different origins (e.g., marine, brackish, and freshwater environments) to eliminate virulent strains and aid in the isolation, identification, and characterization of putative new probiotics.

## Data Availability

The datasets presented in this study can be found in online repositories. The names of the repository/repositories and accession number(s) can be found in the article/Supplementary material.

## Footnotes

1
https://github.com/tseemann/abricate

## Supplementary material

Supplementary Figure S1 The equation as obtained from linear regression on the HHL to estimate the HHL concentration using the diameter of the purple zones on lawns of *Chromobacterium violaceum* strain CV026 after detection of different concentrations of HHL. The HHL concentration was measured in mg.L^–1^.

## References

[B1] Akoll P. Mwanja W. W. (2012). Fish health status, research and management in East Africa: Past and present. *Afr. J. Aquatic Sci.* 37 117–129. 10.2989/16085914.2012.694628 33685377

[B2] Alcock B. P. Huynh W. Chalil R. Smith K. W. Raphenya A. R. Wlodarski M. A. (2023). CARD 2023: Expanded curation, support for machine learning, and resistome prediction at the comprehensive antibiotic resistance database. *Nucleic Acids Res.* 51 D690–D699. 10.1093/nar/gkac920 36263822 PMC9825576

[B3] Amenyogbe E. Zhang J. Huang J. Chen G. (2022). The efficiency of indigenous isolates Bacillus sp. RCS1 and Bacillus cereus RCS3 on growth performance, blood biochemical indices and resistance against Vibrio harveyi in cobia fish (Rachycentron canadum) juveniles. *Aquac. Rep.* 25:101241. 10.1016/j.aqrep.2022.101241

[B4] Azad I. S. Al-Marzouk A. (2008). Autochthonous aquaculture probiotics - A critical analysis. *Res. J. Biotechnol.* 3 171–177.

[B5] Berna M. G. Campa-Córdova, ángel I. Saucedo P. E. González M. C. Marrero R. M. (2015). Isolation and in vitro selection of actinomycetes strains as potential probiotics for aquaculture. *Veterinary World* 8 170–176. 10.14202/vetworld.2015.170-176 27047067 PMC4774698

[B6] Bhunia A. K. (2018). *Food Science Text Series Foodborne Microbial Pathogens.* Berlin: Springer, 10.1007/978-1-4939-7349-1

[B7] Butt U. D. Khan S. Liu X. Sharma A. Zhang X. Wu B. (2024). Present status, limitations, and prospects of using streptomyces bacteria as a potential probiotic agent in aquaculture. *Probiotics Antimicrob. Proteins* 16 426–442. 10.1007/s12602-023-10053-x 36933159 PMC10024021

[B8] Cai Y. Yuan W. Wang S. Guo W. Li A. Wu Y. (2019). In vitro screening of putative probiotics and their dual beneficial effects: To white shrimp (Litopenaeus vannamei) postlarvae and to the rearing water. *Aquaculture* 498 61–71. 10.1016/j.aquaculture.2018.08.024

[B9] Chau K. M. Van T. T. H. Quyen D. V. Le H. D. Phan T. H. T. Ngo N. D. T. (2021). Molecular identification and characterization of probiotic bacillus species with the ability to control vibrio spp. In wild fish intestines and sponges from the vietnam sea. *Microorganisms* 9:1927. 10.3390/microorganisms9091927 34576821 PMC8470590

[B10] Defoirdt T. (2018). Quorum-sensing systems as targets for antivirulence therapy. *Trends Microbiol.* 26 313–328. 10.1016/j.tim.2017.10.005 29132819

[B11] Defoirdt T. Thanh L. D. Van Delsen B. De Schryver P. Sorgeloos P. Boon N. (2011). N-acylhomoserine lactone-degrading Bacillus strains isolated from aquaculture animals. *Aquaculture* 311 258–260. 10.1016/j.aquaculture.2010.11.046

[B12] Deng F. Chen Y. Sun T. Wu Y. Su Y. Liu C. (2021). Antimicrobial resistance, virulence characteristics and genotypes of Bacillus spp. from probiotic products of diverse origins. *Food Res. Int.* 139:109949. 10.1016/j.foodres.2020.109949 33509502

[B13] Deng L. Liu L. Fu T. Li C. Jin N. Zhang H. (2023). Genome sequence and evaluation of safety and probiotic potential of lactiplantibacillus plantarum LPJZ-658. *Microorganisms* 11:11061620. 10.3390/microorganisms11061620 37375122 PMC10301710

[B14] Devescovi G. Kojic M. Covaceuszach S. Cámara M. Williams P. Bertani I. (2017). Negative regulation of violacein biosynthesis in Chromobacterium violaceum. *Front. Microbiol.* 8:349. 10.3389/fmicb.2017.00349 28326068 PMC5339254

[B15] El-Saadony M. T. Alagawany M. Patra A. K. Kar I. Tiwari R. Dawood M. A. O. (2021). The functionality of probiotics in aquaculture: An overview. *Fish Shellf. Immunol.* 117 36–52. 10.1016/j.fsi.2021.07.007 34274422

[B16] Ghanei-Motlagh R. Mohammadian T. Gharibi D. Khosravi M. Mahmoudi E. Zarea M. (2021). Quorum quenching probiotics modulated digestive enzymes activity, growth performance, gut microflora, haemato-biochemical parameters and resistance against Vibrio harveyi in Asian seabass (Lates calcarifer). *Aquaculture* 531:735874. 10.1016/j.aquaculture.2020.735874

[B17] Gopu V. Shetty P. H. (2016). Regulation of acylated homoserine lactones (AHLs) in beef by spice marination. *J. Food Sci. Technol.* 53 2686–2694. 10.1007/s13197-016-2240-x 27478224 PMC4951421

[B18] Goris J. Konstantinidis K. T. Klappenbach J. A. Coenye T. Vandamme P. Tiedje J. M. (2007). DNA-DNA hybridization values and their relationship to whole-genome sequence similarities. *Int. J. Syst. Evol. Microbiol.* 57 81–91. 10.1099/ijs.0.64483-0 17220447

[B19] Hasan K. N. Banerjee G. (2020). Recent studies on probiotics as beneficial mediator in aquaculture: A review. *J. Basic Appl. Zool.* 81 53. 10.1186/s41936-020-00190-y

[B20] Hossain T. J. Das M. Ali F. Chowdhury S. I. Zedny S. A. (2021). Substrate preferences, phylogenetic and biochemical properties of proteolytic bacteria present in the digestive tract of Nile tilapia (Oreochromis niloticus). *AIMS Microbiol.* 7 528–545. 10.3934/microbiol.2021032 35071947 PMC8712536

[B21] Husain F. Duraisamy S. Balakrishnan S. Ranjith S. Chidambaram P. Kumarasamy A. (2022). Phenotypic assessment of safety and probiotic potential of native isolates from marine fish Moolgarda seheli towards sustainable aquaculture. *Biologia* 77 775–790. 10.1007/s11756-021-00957-w 35034969 PMC8744026

[B22] Jinendiran S. Boopathi S. Sivakumar N. Selvakumar G. (2019). Functional characterization of probiotic potential of novel pigmented bacterial strains for aquaculture applications. *Probiotics Antimicrob. Proteins* 11 186–197. 10.1007/s12602-017-9353-z 29181803

[B23] Jolley K. A. Bliss C. M. Bennett J. S. Bratcher H. B. Brehony C. Colles F. M. (2012). Ribosomal multilocus sequence typing: Universal characterization of bacteria from domain to strain. *Microbiology* 158 1005–1015. 10.1099/mic.0.055459-0 22282518 PMC3492749

[B24] Kato C. Mugaanyi M. Majalija S. Tamale A. Musisi N. Sengooba A. (2016). Isolation and identification of potential probiotics bacteria from the gut of oreochromis niloticus and Clarias gariepinus in Uganda. *Br. Microbiol. Res. J.* 17 1–8. 10.9734/bmrj/2016/29721

[B25] Kaushik J. K. Kumar A. Duary R. K. Mohanty A. K. Grover S. Batish V. K. (2009). Functional and probiotic attributes of an indigenous isolate of lactobacillus plantarum. *PLoS One* 4:e0008099. 10.1371/journal.pone.0008099 19956615 PMC2779496

[B26] Kavitha M. Raja M. Perumal P. (2018). Evaluation of probiotic potential of Bacillus spp. isolated from the digestive tract of freshwater fish Labeo calbasu (Hamilton, 1822). *Aquac. Rep.* 11 59–69. 10.1016/j.aqrep.2018.07.001

[B27] Khan K. U. Rodrigues A. T. Mansano C. F. M. Queiroz D. M. A. Sakomura N. K. Romaneli R. S. (2019). Dietary protein quality and proper protein to energy ratios: A bioeconomic approach in aquaculture feeding practices. *Latin Am. J. Aquatic Res.* 47 232–239.

[B28] Knipe H. Temperton B. Lange A. Bass D. Tyler C. R. (2021). Probiotics and competitive exclusion of pathogens in shrimp aquaculture. *Rev. Aquac.* 13 324–352. 10.1111/raq.12477

[B29] Kroll J. Klinter S. Schneider C. Voß I. Steinbüchel A. (2010). Plasmid addiction systems: Perspectives and applications in biotechnology. *Microbial Biotechnol.* 3 634–657. 10.1111/j.1751-7915.2010.00170.x 21255361 PMC3815339

[B30] Kuebutornye F. K. A. Abarike E. D. Lu Y. (2019). A review on the application of Bacillus as probiotics in aquaculture. *Fish Shellf. Immunol.* 87 820–828. 10.1016/j.fsi.2019.02.010 30779995

[B31] Kuebutornye F. K. A. Abarike E. D. Lu Y. Hlordzi V. Sakyi M. E. Afriyie G. (2020). Mechanisms and the role of probiotic Bacillus in mitigating fish pathogens in aquaculture. *Fish Physiol. Biochem.* 46 819–841. 10.1007/s10695-019-00754-y 31953625

[B32] Kuebutornye F. K. A. Lu Y. Abarike E. D. Wang Z. Li Y. Sakyi M. E. (2020). In vitro assessment of the probiotic characteristics of three bacillus species from the gut of nile tilapia, oreochromis niloticus. *Probiotics Antimicrob. Proteins* 12 412–424. 10.1007/s12602-019-09562-5 31243734

[B33] LaSarre B. Federle M. J. (2013). Exploiting quorum sensing to confuse bacterial pathogens. *Microbiol. Mol. Biol. Rev.* 77 73–111. 10.1128/mmbr.00046-12 23471618 PMC3591984

[B34] Liu B. Zheng D. Jin Q. Chen L. Yang J. (2019). VFDB 2019: A comparative pathogenomic platform with an interactive web interface. *Nucleic Acids Res.* 47 D687–D692. 10.1093/nar/gky1080 30395255 PMC6324032

[B35] Liu C. H. Chiu C. S. Ho P. L. Wang S. W. (2009). Improvement in the growth performance of white shrimp, Litopenaeus vannamei, by a protease-producing probiotic, Bacillus subtilis E20, from natto. *J. Appl. Microbiol.* 107 1031–1041. 10.1111/j.1365-2672.2009.04284.x 19320951

[B36] Liu Y. Y. Hsu C. Y. Yang Y. C. Huang C. H. Chen C. C. (2023). ProbioMinServer: An integrated platform for assessing the safety and functional properties of potential probiotic strains. *Bioinformatics Adv.* 3:vbad153. 10.1093/bioadv/vbad153 37928343 PMC10625473

[B37] Lu S. Na K. Li Y. Zhang L. Fang Y. Guo X. (2022). Bacillus-derived probiotics: Metabolites and mechanisms involved in bacteria–host interactions. *Crit. Rev. Food Sci. Nutr.* 64 1701–1714. 10.1080/10408398.2022.2118659 36066454

[B38] Luu-Thi H. Khadka D. B. Michiels C. W. (2014). Thermal inactivation parameters of spores from different phylogenetic groups of Bacillus cereus. *Int. J. Food Microbiol.* 189 183–188. 10.1016/j.ijfoodmicro.2014.07.027 25171111

[B39] Mathan Muthu C. M. Vickram A. S. Bhavani Sowndharya B. Saravanan A. Kamalesh R. Dinakarkumar Y. (2024). A comprehensive review on the utilization of probiotics in aquaculture towards sustainable shrimp farming. *Fish Shellf. Immunol.* 147:109459. 10.1016/j.fsi.2024.109459 38369068

[B40] Mbowa S. Odokonyero T. Munyaho A. (2017). Harnessing floating cage technology to increase fish production in Uganda. *Res. Ser.* 262886:44.

[B41] Meier-Kolthoff J. P. Göker M. (2019). TYGS is an automated high-throughput platform for state-of-the-art genome-based taxonomy. *Nat. Commun.* 10:2182. 10.1038/s41467-019-10210-3 31097708 PMC6522516

[B42] Mukherjee A. Dutta D. Banerjee S. Ringø E. Breines E. M. Hareide E. (2017). Culturable autochthonous gut bacteria in rohu, Labeo rohita. In vitro growth inhibition against pathogenic Aeromonas spp., stability in gut, bio-safety and identification by 16S rRNA gene sequencing. *Symbiosis* 73 165–177. 10.1007/s13199-017-0474-7

[B43] Naigaga S. (2018). *Potential Influence of Climate Variations, Water Quality and Soil Quality on Uganda’s Aquaculture.* Available online at: https://etd.auburn.edu/bitstream/handle/10415/6425/Shamim%20Naigaga%20Dissertation.pdf?sequence=2

[B44] Nayak S. K. (2010). Probiotics and immunity: A fish perspective. *Fish Shellf. Immunol.* 29 2–14. 10.1016/j.fsi.2010.02.017 20219683

[B45] Nemutanzhela M. E. Roets Y. Gardiner N. Lalloo R. (2014). *The Use and Benefits of Bacillus Based Biological Agents in Aquaculture.* London: Intechopen.

[B46] Paray A. A. Singh M. Amin Mir M. (2023). Gram staining: A Brief review. *Int. J. Res. Rev.* 10 336–341. 10.52403/ijrr.20230934

[B47] Roy S. Kumar V. Bossier P. Norouzitallab P. Vanrompay D. (2019). Phloroglucinol treatment induces transgenerational epigenetic inherited resistance against vibrio infections and thermal stress in a brine shrimp (Artemia franciscana) model. *Front. Immunol.* 10:2745. 10.3389/fimmu.2019.02745 31827471 PMC6890837

[B48] Sarkar P. Issac P. K. Raju S. V. Elumalai P. Arshad A. Arockiaraj J. (2021). Pathogenic bacterial toxins and virulence influences in cultivable fish. *Aquac. Res.* 52 2361–2376. 10.1111/are.15089

[B49] Serwecińska L. (2020). Antimicrobials and antibiotic-resistant bacteria: A risk to the environment and to public health. *Water* 12:3313. 10.3390/w12123313

[B50] Sun Y. Li H. Zheng L. Li J. Hong Y. Liang P. (2022). iProbiotics: A machine learning platform for rapid identification of probiotic properties from whole-genome primary sequences. *Brief. Bioinf.* 23:bbab477. 10.1093/bib/bbab477 34849572

[B51] Tinh N. T. N. Asanka Gunasekara R. A. Y. S. Boon N. Dierckens K. Sorgeloos P. Bossier P. (2007). N-acyl homoserine lactone-degrading microbial enrichment cultures isolated from Penaeus vannamei shrimp gut and their probiotic properties in Brachionus plicatilis cultures. *FEMS Microbiol. Ecol.* 62 45–53. 10.1111/j.1574-6941.2007.00378.x 17784866

[B52] Vandeputte M. Coppens S. Bossier P. Vereecke N. Vanrompay D. (2024). Genomic mining of Vibrio parahaemolyticus highlights prevalence of antimicrobial resistance genes and new genetic markers associated with AHPND and tdh + /trh + genotypes. *BMC Genomics* 25:178. 10.1186/s12864-024-10093-9 38355437 PMC10868097

[B53] Vereecke N. Van Hoorde S. Sperling D. Theuns S. Devriendt B. Cox E. (2023). Virotyping and genetic antimicrobial susceptibility testing of porcine ETEC/STEC strains and associated plasmid types. *Front. Microbiol.* 14:1139312. 10.3389/fmicb.2023.1139312 37143544 PMC10151945

[B54] Villegas-Plazas M. Villamil L. Martínez-Silva M. A. González-Jiménez T. Salazar M. Güiza L. (2022). Microbiome composition and autochthonous probiotics from contrasting probiosis/dysbiosis states in cobia (Rachycentron canadum) fish epitheliocystis. *Access Microbiol.* 4:405. 10.1099/acmi.0.000405 36133177 PMC9484664

[B55] Visiello R. Colombo S. Carretto E. (2016). Bacillus cereus hemolysins and other virulence factors. *Diverse Faces Bacillus Cereus* 29 35–44. 10.1016/B978-0-12-801474-5.00003-7

[B56] Walakira J. Akoll P. Engole M. Sserwadda M. Nkambo M. Namulawa V. (2014). Common fish diseases and parasites affecting wild and farmed Tilapia and catfish in Central and Western Uganda. *Uganda J. Agric. Sci.* 15 113–125.

[B57] Wang M. Yi M. Lu M. Gao F. Liu Z. Huang Q. (2020). Effects of probiotics Bacillus cereus NY5 and Alcaligenes faecalis Y311 used as water additives on the microbiota and immune enzyme activities in three mucosal tissues in Nile tilapia Oreochromis niloticus reared in outdoor tanks. *Aquac. Rep.* 17:100309. 10.1016/j.aqrep.2020.100309

[B58] Yamashita M. M. Ferrarezi J. V. Pereira G. do V. Bandeira G. Côrrea (2020). Autochthonous vs allochthonous probiotic strains to Rhamdia quelen. *Microbial Pathog.* 139:103897. 10.1016/j.micpath.2019.103897 31786258

[B59] Yoon S. H. Ha S. Lim J. Kwon S. Chun J. (2017). A large-scale evaluation of algorithms to calculate average nucleotide identity. *Antonie van Leeuwenhoek Int. J. General Mol. Microbiol.* 110 1281–1286. 10.1007/s10482-017-0844-4 28204908

[B60] Yousuf S. Tyagi A. Singh R. (2023). Probiotic supplementation as an emerging alternative to chemical therapeutics in finfish aquaculture: A review. *Probiotics Antimicrob. Proteins* 15 1151–1168. 10.1007/s12602-022-09971-z 35904730

