# Supplementary figures and images for: Advancing aquaculture probiotic discovery via an innovative protocol for isolation of indigenous, heat and salt tolerant, quorum quenching probiotic candidates
Source: Front Microbiol. 2025 Apr 2;16:1558238. doi: 10.3389/fmicb.2025.1558238 (PMC12000936; doi:10.3389/fmicb.2025.1558238)

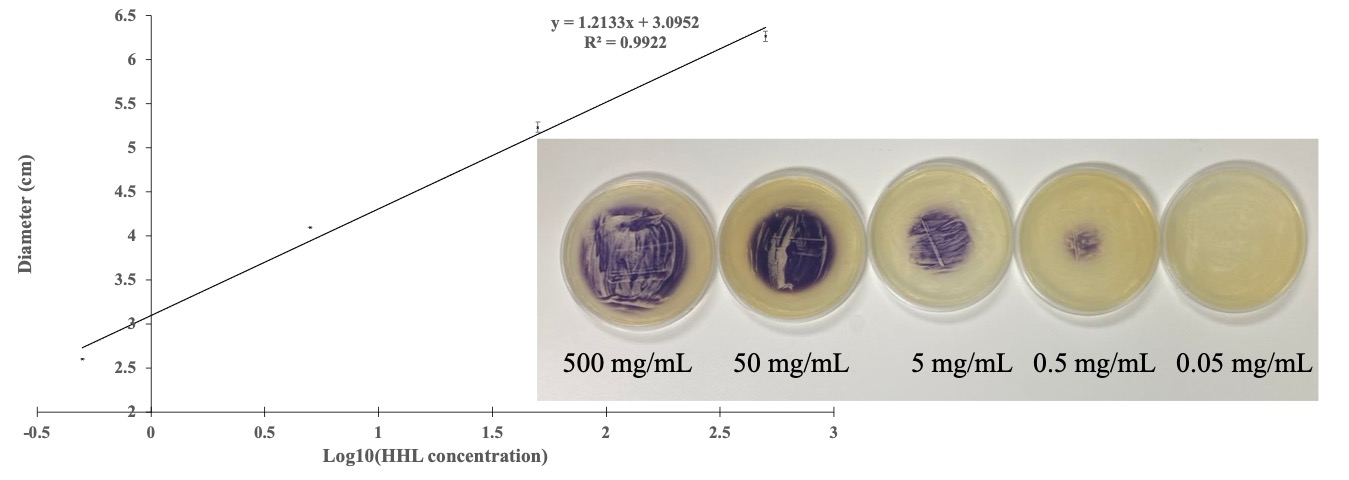

Supplement: Supplementary Figure S1 — The equation as obtained from linear regression on the HHL to estimate the HHL concentration using the diameter of the purple zones on lawns of Chromobacterium violaceum strain CV026 after detection of different concentrations of HHL. The HHL concentration was measured in mg.L–1. [file Image_1.jpeg]
